# Seasonal cues induce phenotypic plasticity of *Drosophila suzukii* to enhance winter survival

**DOI:** 10.1186/s12898-016-0070-3

**Published:** 2016-03-22

**Authors:** Peter W. Shearer, Jessica D. West, Vaughn M. Walton, Preston H. Brown, Nicolas Svetec, Joanna C. Chiu

**Affiliations:** Mid-Columbia Agricultural Research and Extension Center, Oregon State University, 3005 Experiment Station Drive, Hood River, OR 97331 USA; Department of Entomology and Nematology, University of California, Davis, CA 95616 USA; Department of Horticulture, Oregon State University, Corvallis, OR 97331 USA; Department of Evolution and Ecology, University of California, Davis, CA 95616 USA

**Keywords:** *Drosophila suzukii*, Phenotypic plasticity, Cold tolerance, Diapause, High-throughput sequencing, Transcriptome

## Abstract

**Background:**

As global climate change and exponential human population growth intensifies pressure on agricultural systems, the need to effectively manage invasive insect pests is becoming increasingly important to global food security*. Drosophila suzukii* is an invasive pest that drastically expanded its global range in a very short time since 2008, spreading to most areas in North America and many countries in Europe and South America. Preliminary ecological modeling predicted a more restricted distribution and, for this reason, the invasion of *D. suzukii* to northern temperate regions is especially unexpected. Investigating *D. suzukii* phenology and seasonal adaptations can lead to a better understanding of the mechanisms through which insects express phenotypic plasticity, which likely enables invasive species to successfully colonize a wide range of environments.

**Results:**

We describe seasonal phenotypic plasticity in field populations of *D. suzukii*. Specifically, we observed a trend of higher proportions of flies with the winter morph phenotype, characterized by darker pigmentation and longer wing length, as summer progresses to winter. A laboratory-simulated winter photoperiod and temperature (12:12 L:D and 10 °C) were sufficient to induce the winter morph phenotype in *D. suzukii.* This winter morph is associated with increased survival at 1 °C when compared to the summer morph, thus explaining the ability of *D. suzukii* to survive cold winters. We then used RNA sequencing to identify gene expression differences underlying seasonal differences in *D. suzukii* physiology. Winter morph gene expression is consistent with known mechanisms of cold-hardening such as adjustments to ion transport and up-regulation of carbohydrate metabolism. In addition, transcripts involved in oogenesis and DNA replication were down-regulated in the winter morph, providing the first molecular evidence of a reproductive diapause in *D. suzukii*.

**Conclusions:**

To date, *D. suzukii* cold resistance studies suggest that this species cannot overwinter in northern locations, e.g. Canada, even though they are established pests in these regions. Combining physiological investigations with RNA sequencing, we present potential mechanisms by which *D. suzukii* can overwinter in these regions. This work may contribute to more accurate population models that incorporate seasonal variation in physiological parameters, leading to development of better management strategies.

**Electronic supplementary material:**

The online version of this article (doi:10.1186/s12898-016-0070-3) contains supplementary material, which is available to authorized users.

## Background

It is estimated that insects account for 18 % of global crop production losses [1]. An increase in average global temperature will likely intensify the damage caused by insect pests, as higher average temperature is predicted to increase insect populations through greater overwintering survival, higher reproductive rates, and an increased number of generations [2, 3]. In particular, invasive species may have an advantage over indigenous species in such conditions [4–6]. Therefore, it is imperative to understand how invasive species can successfully invade and compromise ecosystems, sometimes in a very short timeframe. It has been hypothesized that high levels of phenotypic plasticity play an important role in the success of invasive species in changing conditions, such as those caused by global climate change [6, 7].

One such invasive species, *Drosophila suzukii* Matsumura (Diptera: Drosophilidae) was first discovered in the continental USA (Watsonville, CA) in 2008 [8] and has rapidly spread to become an established pest of fruit crops all over the world, including North and South America and much of Europe, [9–13]. Commonly known as the Spotted Wing Drosophila, this vinegar fly has an enlarged, serrated ovipositor, allowing adult females to penetrate the skin of soft-skinned, ripening fruit and lay eggs inside, where the larvae feed and destroy the fruit [11]. *Drosophila suzukii* most commonly infests cherries, blackberries, raspberries, and strawberries, but has also been found to oviposit into grapes, plums, peaches, and other fruits [11]. In the U.S. alone, *D. suzukii* invasions have caused significant crop losses, and costs directly related to management practices are estimated to vary between $129 and 172 million (6–8 % of farmgate value) annually [14].

*Drosophila suzukii* has a wide climatic presence, causing economic losses of affected fruits in areas ranging from mild subtropical production regions to severe continental climates [15]. In Asia, where this species is native, *D. suzukii* are preferentially found at higher altitudes and higher latitudes when compared to other closely related species [16]. Previous studies conducted on *D. suzukii* cold tolerance predict that this species will likely not survive extended periods of cold such as those found in production regions in Canada, Eastern Oregon, Washington, and Michigan [17]. Despite these predictions, *D. suzukii* is now an established pest in those regions [12, 15, 18], and in fact has proven successful in a wide range of environments ranging from Southern California to British Columbia, Canada [13], raising the question of how this species can adapt to the harsh climates in more northern locations.

Insects exhibit a wide variety of strategies to increase cold tolerance and overwinter. There are two main classes of cold-hardening: (1) seasonal cold-hardening, which is induced over a timescale of days to weeks, and (2) rapid cold-hardening, which can occur in minutes or hours, and is induced by a sudden drop in temperature like a cold snap [19]. Both seasonal and rapid cold-hardening mechanisms include adjustments to ion transport and membrane restructuring to increase membrane fluidity at low temperatures. The synthesis of cryoprotectants, typically polyols such as glycerol, sorbitol, or inositol, is an important mechanism in seasonal cold-hardening, but it is unclear whether it is associated with rapid cold-hardening. Up-regulation of antifreeze proteins and ice nucleating agents are mechanisms that increase cold tolerance in seasonally cold-hardened insects, but are not associated with rapid cold-hardening. Inhibition of apoptotic cell death, MAP kinase signaling, and calcium signaling have all been found to be important mechanisms of the rapid cold-hardening response, but have not been found to occur in seasonal cold-hardening [19].

Seasonal cold-hardening is often an essential component of winter diapause, a process characterized by developmental arrest, decreased metabolic activity, and a general state of dormancy [19–21]. Reproductive diapause is an adaptation that allows an organism to temporarily cease reproduction in order to conserve resources to survive unfavorable conditions, continuing reproduction when more favorable conditions arise [22]. In fact, evolution of diapause has been linked to enabling range expansion in other invasive species [23, 24]. Diapause is found to occur in many *Drosophila* species, including *D. melanogaster*, yet can vary significantly among clinally distributed natural populations [25–28]. In *D. suzukii*, high rates of reproductively immature adult females have been observed in the months leading up to winter in Hokkaido, Japan, suggesting that a reproductive diapause may occur in this species [29]. Additionally, *D. suzukii* has a relatively low nucleotide substitution rate when compared to other Drosophilids [16]. This is consistent with presence of a reproductive diapause in this species, as a low substitution rate may be caused by fewer generations per year. Diapause incidence has also been assessed via ovary dissection (Anna K. Wallingford, Jana C. Lee, Gregory M. Loeb, personal communications). Wallingford et al. found that at a photoperiod of 12:12 L:D and 10 °C, there were almost no reproductively mature females in laboratory conditions, and no reproductively mature females in December at field collection sites in Oregon and New York.

In addition to undergoing reproductive diapause, Drosophilids are known to exhibit multiple strategies to survive suboptimal cold temperatures and low humidity. These strategies include accumulation of cryprotectants such as maltose, trehalose, proline, and myo-inositol [30–34], altered composition of membrane phospholipids [30, 35], and increased expression of stress-induced genes such as heat shock proteins [36–38]. Darker cuticle pigmentation has been hypothesized to be involved in thermoregulation of ectotherms in cold environments, resulting in increased ultraviolet absorption and increased ability to warm up [39, 40]. However, increased melanization has also been implicated in immunity and increased desiccation resistance [41–43]. A larger body size in colder environments may also be advantageous in colder temperatures, as it may be involved in thermoregulation [44–46] or cost-benefits of altered membrane fluidity [45], but the relationship between body size and temperature is not well understood [47, 48]. Latitudinal clines of adult body size are known to exist for many ectotherms, including several *Drosophila* species [49, 50], with smaller flies observed in warmer places, but this phenomenon is not well conserved in insects and other ectotherms [47, 51]. In addition to the inverse relationship of temperature and body size observed in natural environments, most ectotherms, including *D. melanogaster*, grow to be smaller sizes when raised in warmer temperatures in the laboratory [45, 47, 48].

Phenotypic plasticity is a phenomenon by which one genotype can lead to multiple phenotypes in different environmental conditions [52]. Phenotypic plasticity often occurs in response to seasonal changes in order for the insect to display traits that best suit seasonal conditions, producing a seasonal morph [22]. In some cases, seasonal morphs are tightly linked to diapause [22]. A recent study reported on *D. suzukii* seasonal morphs [53], in which they found that *D. suzukii* winter morphs are able to survive lower temperatures than *D. suzukii* summer morphs, helping to explain the wide climatic presence of this invasive pest. In this study, we characterized seasonal phenotypic plasticity in *D. suzukii* in both field-collected populations and flies placed under simulated seasonal conditions in the laboratory. We quantified phenotypic characters and measured differences in low-temperature survival rates between the summer and winter morphs of *D. suzukii*. Finally, we used RNA sequencing to examine transcriptomic differences that underlie the observed phenotypic divergence between the adult stages of the two seasonal morphs. Knowledge of *D. suzukii* survival strategies in the winter is essential and of great economic importance, as it will direct the development of more effective management strategies.

## Results

### *D. suzukii* exhibit seasonal variations in phenological traits in the field

**Fig. 1:**
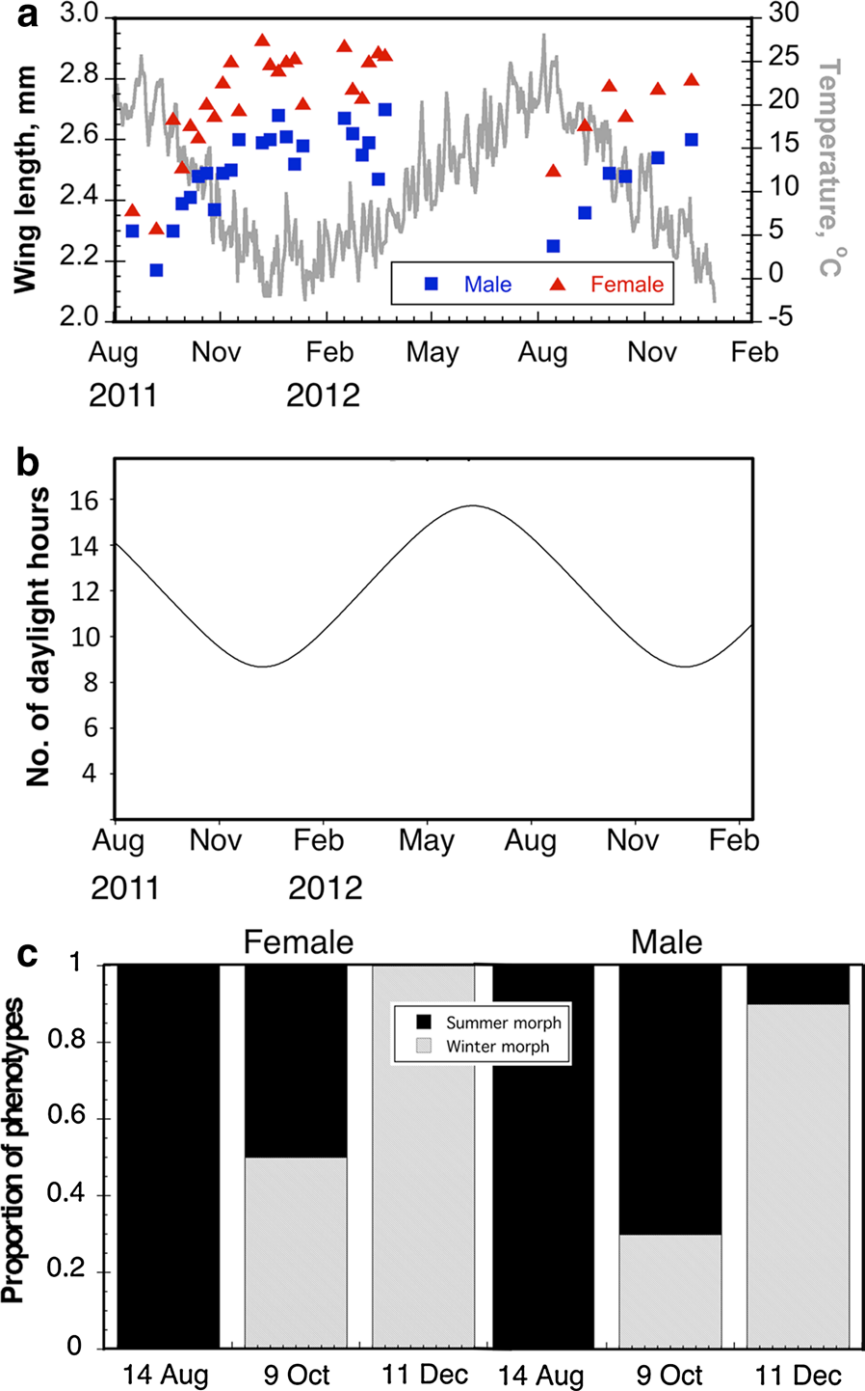
*Drosophila suzukii* exhibit phenotypic plasticity in size and melanization in the field. **a** Seasonal changes in wing length of female *D. suzukii* collected from the field (Hood River, OR, USA) in apple cider vinegar baited traps. Both wing length (mm) (*left axis*) and temperature (°C) (*right axis*) are represented on the Y-axes, and chronological time (between August 2011 to February 2013) is represented on the *X-axis*. Male wing length is plotted in *blue* and female wing length is plotted in *red*. Temperature is plotted in *gray*. Mean daily air temperatures are plotted from the Hood River, Oregon AgriMet Weather station (HOXO) [73]. **b** Seasonal change in photoperiod from August 2011 to February 2013. The day-length (in hours) is shown on the *Y-axis* and date is shown on the *X-axis*. Day-length values were *plotted* for Hood River, Oregon [74]. **c** Percent summer morph (lighter pigmentation) and winter morph (*darker pigmentation*) of female (*left*) and male (*right*) *D. suzukii* from August to December in Hood River, OR during 2011. Summer and winter morphs are represented by *black* and *gray* respectively

Female *D. suzukii* collected seasonally from traps located in Hood River, OR, USA displayed large variation in individual body size throughout the 2011 and 2012 samples (Fig. 1a). To determine the possible factors underlying seasonal variation in body size, we monitored two abiotic factors: temperature of development and day-length. As proxy for developmental temperature, we used the average temperature for the 12-day period preceding the collection date. To ascertain whether there is a significant relationship between these factors and wing length, we then performed, for each sex, linear regressions of wing length (i.e. a proxy of body size) over temperature and day-length. The 12-day mean temperature explained 68 % (*R*^*2*^ = 0.68) of the observed variation in wing length for females where it explained 77 % (*R*^*2*^ = 0.68) of the observed variation for male wing size (both linear regressions: *P* < 0.0001). The mean wing length was also negatively correlated to day-length (Fig. 1b) Day-length explained 40 % (*R*^*2*^ = 0.40) of the observed variation in female wing length, and 47 % (*R*^2^ = 0.4658) of the observed variation in males’ wing length (both linear regressions: *P* < 0.0001). Moreover, the seasonal composition of both male and female *D. suzukii* winter morph increased from levels of 0 % of both sexes to 100 and 95 % respectively when examining dates starting on 14 August to 11 December, 2011 (Fig. 1c).

### Simulated summer and winter conditions in the laboratory induce differences in phenological traits

**Fig. 2:**
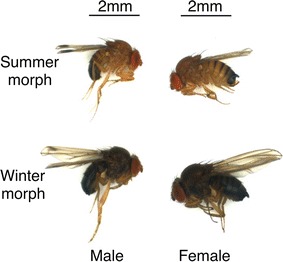
Phenotypic variation of laboratory-reared *D. suzukii* expressed by different photoperiod and temperature regimes. Summer morph adults are reared at 20 °C and 16:8 L:D photoperiod (*top panels*); winter morph adults are reared at 10 °C and 12:12 L:D photoperiod (*bottom panels*)

To investigate the impact of environmental factors in influencing seasonal variations in *D. suzukii* morphology and physiology, we tested effects of simulated summer and winter laboratory conditions by varying photoperiod and temperature. Differences in adult *D. suzukii* body color and wing length reared at 16:8 Light:Dark (L:D) in hours and 20 °C as compared to 12:12 L:D and 10 °C were visually apparent (Fig. 2), and closely resemble summer and winter morphs that we observed in the field. Abdominal melanization ratings were significantly higher for fourth abdominal segments of female flies subjected to 12:12 L:D and 10 °C compared to female flies that were reared at 16:8 L:D and 20 °C (t = −20.6; df = 16; *P* < 0.0001) (Table 1). Similarly, abdominal melanization ratings were significantly higher for the third abdominal segments for male flies that were reared in 12:12 L:D and 10 °C when compared to male flies that were housed in 16:8 L:D and 20 °C (t = −13.5; df = 27; *P* < 0.0001) (Table 1).

**Table 1:** Average melanization rating of dorsal abdominal bands of female and male *Drosophila suzukii* seasonal morphs

Seasonal morph and sex^b^	Melanization rating^a^				
	Abdominal segment				
	1st	2nd	3rd	4th	5th
Summer female	1.1	2.0	1.9	1.8^*, c^	4.6
Winter female	2.8	2.9	3.5	5.0	5.0
Summer male	1.4	2.1	2.6^*^	5.0	5.0
Winter male	3.5	3.6	4.9	5.0	5.0

n = 13 for winter male and female; n = 17 for summer male and female

^a^Melanization rating based on visual rating of the thickness of the transverse dark line of each dorsal abdominal segment (Additional file 1: Figure S1): *1* thin dark line, *5* completely dark

^b^Summer morphs were reared at 16:8 L:D and 20 °C; winter morphs were reared at 12:12 L:D and 10 °C

^c^Means followed by an asterisk are significantly different within a sex (t test, P ≤ 0.05)

We then conducted a series of experiments to examine intergeneration effects of photoperiod and temperature on wing length. We first examined the effect of photoperiod alone on wing length (Table 2). Holding temperature constant at 20 °C, we either kept parent flies (F0), which were reared in 16:8 L:D photoperiod, in the same photoperiod (16:8 L:D) or transferred the adult parent flies (F0) to 12:12 L:D and examine the resulting offsprings (F1). Not surprisingly, there was no difference in wing length for female offsprings when parents were maintained in a photoperiod of 16:8 L:D as compared to their female parents (Table 2). However, when the parents were transferred to 12:12 L:D, their female offsprings displayed significantly increased wing length compared to female offsprings with parents reared under 16:8 L:D (*F* = 37.7; df = 2, 32; *P* < 0.0001) (Table 2).

**Table 2:** Effect of photoperiod on female *Drosophila suzukii* wing length over two generations

Environmental parameters				Wing length (mm)^a^
Parental (F0)		Offspring (F1)		
Day-length (L:D)	Temp (°C)	Day-length (L:D)	Temp (°C)	
16:8	20			2.2 b
		16:8	20	2.2 b
		12:12	20	2.5 a

^a^Mean values of wing length followed by the same letter are not significantly different, ANOVA, Tukey. n = 5 for each mean

We next examined the effect of both photoperiod and temperature on wing length (Table 3). In a photoperiod of 16:8 L:D, if parents (F0) were transferred from 20 to 10 °C, their female offsprings had significantly increased wing length when compared to their parents, which were originally raised in 20 °C (*F* = 215.9; df = 2, 33; *P* < 0.0001) (Table 3). However, compounding the change in temperature (from 20 to 10 °C) with a shortening of day-length (16–12 h) did not further affect wing length of female offsprings (Table 3).

**Table 3:** Effect of photoperiod and temperature on female *Drosophila suzukii* wing length over two generations

Environmental parameters				Wing length (mm)^a^
Parental (F0)		Offspring (F1)		
Day-length (L:D)	Temp (°C)	Day-length (L:D)	Temp (°C)	
16:8	20			2.4 b
		16:8	10	2.9 a
		12:12	10	2.9 a

^a^Mean values of wing length followed by the same letter are not significantly different, ANOVA, Tukey. n = 5 for each mean

We further investigated the effect of shortening day-length and decrease in temperature on female *D. suzukii* wing length and melanization (Table 4). When parent flies (F0), which were originally reared in 16:8 L:D and 20 °C, were transferred to a photoperiod of 12:12 L:D either at 10 or 20 °C, the average wing length of their offsprings were significantly longer as compared to their parents. Offsprings produced under 12:12 L:D and 10 °C had the largest wings (*F* = 134.31; df = 2, 38; *P* < 0.0001 (Table 4). The melanization rating of the fourth abdominal segment was greater for female offsprings produced at 10 °C and 12:12 L:D than at 20 °C and 12:12 L:D, while the width of the bands of their female parents, which were originally produced at 16:8 L:D and 20 °C, were intermediate compared with band width of their offspring (*F* = 48.38; df = 2, 42; *P* < 0.0001 with regard to melanization ratings).

**Table 4:** Effect of shortening day-length and decrease in temperature on female *Drosophila suzukii* wing length and abdominal melanization over two generations

Environmental parameters				Wing length (mm)^a^	Melanization rating of segment 4^a^
Parental (F0)		Offspring (F1)			
Day-length (L:D)	Temp (°C)	Day-length (L:D)	Temp (°C)		
16:8	20			2.1 c	3.1 b
		12:12	10	2.9 a	4.8 a
		12:12	20	2.5 b	2.2 c

^a^Mean values within a column followed by the same letter are not significantly different, ANOVA: Wing length: F = 134.31; df = 2, 38; *P* < 0.0001. Melanization rating: *F* = 48.38; df = 2, 42; *P* < 0.0001. Tukey. n = 5 for each mean

Our results point to a complex interaction between photoperiod and temperature in affecting wing length and abdominal melanization. Although either shorter day-length or lower temperature can independently induce increase in wing length (Tables 2, 3), transition from long to short day-length did not appear to provide added positive effect on increased wing length if it is accompanied by a decrease in temperature (Table 3). Interestingly, transition from summer-like (20 °C) to winter-like (10 °C) temperature showed an additive effect if accompanied by a decrease in day-length (Table 4). Unlike wing length, which increases in response to changes that signal winter (short day-length and lower temperature), abdominal melanization appeared to be differentially regulated by these two cues that signal winter: shorter day-length decreased the melanization while lower temperature greatly increased abdominal melanization (Table 4).

### Survival of summer and winter morphs at different temperatures

**Fig. 3:**
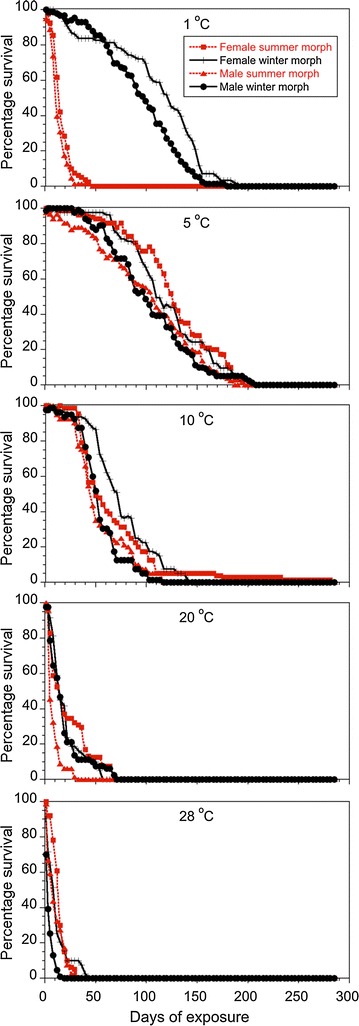
Mortality curves for summer and winter morphs of *D. suzukii* at five controlled temperatures. Summer and winter morphs of *D. suzukii* (male and female adults) were maintained at 1, 5, 10, 20, and 28 °C, and their survival were assessed

**Fig. 4:**
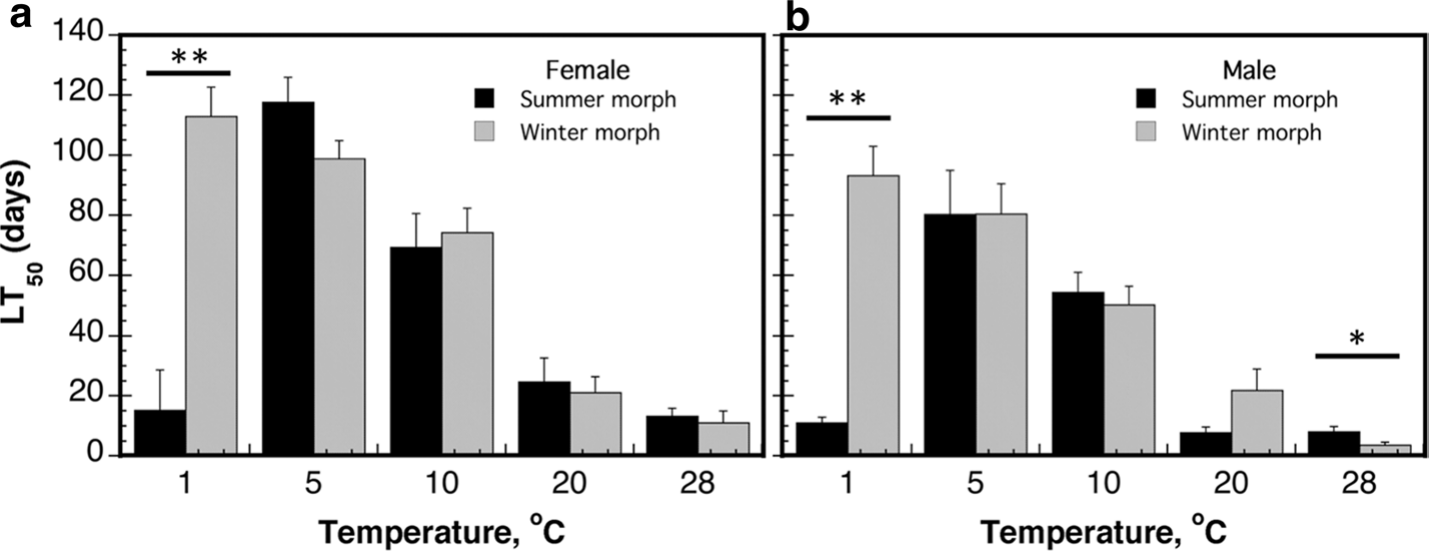
Length of time (days) for female and male morphs to reach 50 % mortality (LT_50_) at various constant temperatures. LT_50_ was calculated for the summer and winter morphs of (**a**) female *D. suzukii*, and (**b**) male *D. suzukii* when maintained at 1, 5, 10, 20, and 28 °C. Paired t tests were performed on estimated LT_50_ values (days) for each sex between summer and winter morph at each temperature. Significant differences were indicated by *asterisks*. (* indicates *P* < 0.05 and ** indicates *P* < 0.01)

To examine whether a transition to winter morphs provided a survival advantage in winter conditions, specifically low temperature, we subjected summer and winter morphs of *D. suzukii* to various temperature conditions (1, 5, 10, 20 and 28 °C) and measured their survival rates (Fig. 3). Paired t tests performed on estimated LT_50_ values (days) for each sex at each temperature revealed that adult female winter morph *D. suzukii* lived significantly longer than adult female summer morph (LT_50_ = 115 vs. LT_50_ = 28 d, respectively) at 1 °C (t = −6.36; df = 7; *P* = 0.0004) (Figs. 3, 4). No other statistical differences in LT_50_ survival between female morphs were observed at the four other temperature regimes (t = −0.46 to 2.35; df = 7; *P* = 0.07–0.95). Male winter morph *D. suzukii* had a higher LT_50_ value than male summer morph *D. suzukii* (LT_50_ = 93 vs. LT_50_ = 11 d, respectively) at 1 °C (t = −9.37; df = 7; *P* < 0.0001) (Figs. 3, 4). Conversely, male summer morph *D. suzukii* survived longer at 28 °C than male winter morph (LT_50_ = 8 vs. LT_50_ = 3 d, respectively) (t = 2.72; df = 7; *P* = 0.03). No other statistical differences in survival time between male morphs were observed at the other three temperature regimes (t = −1.74 to 0.02; df = 7; *P* = 0.13–0.98).

We also tested for sex-specific differences in survival at 1 °C. There was a significant difference in the LT_50_ values for male (mean = 93.1 d; SEM = 9.8) and female (mean = 112.9 d; SEM = 9.8) winter morphs held at 1 °C (t = 2.114; df = 7; *P* = 0.036). In this instance, the LT_50_ for females was approximately 20 days longer than for males.

### Gene expression differences in summer and winter morph

**Fig. 5:**
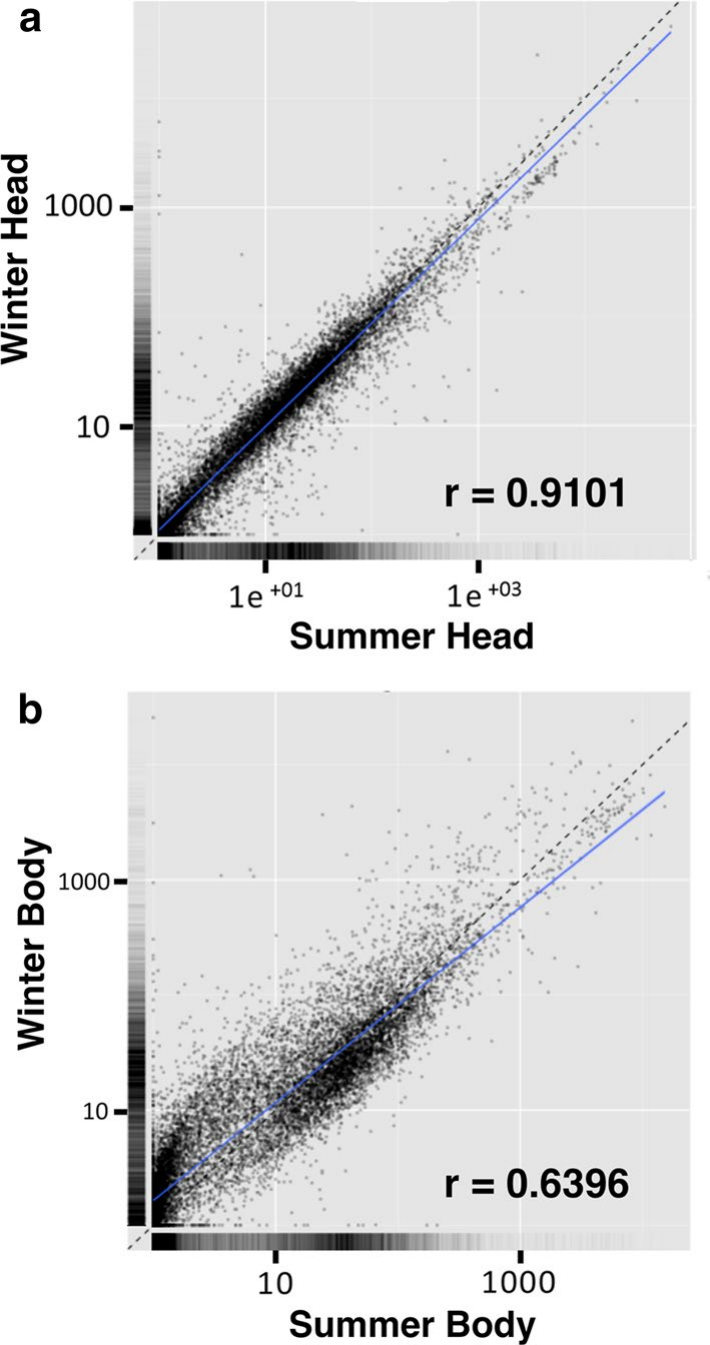
Correlation of gene expression between summer and winter morphs of *D. suzukii*. The FPKM values for all transcripts were plotted for summer and winter morphs by averaging across the biological replicates. Pairwise FPKM comparisons were generated using the csScatter() function from CummeRbund in (**a**) heads and (**b**) bodies. Pearson’s correlation coefficient were calculated using the cor() function in R. *Dotted line* represents r = 1. *Solid line* represents deduced “r” value as calculated using the data

**Fig. 6:**
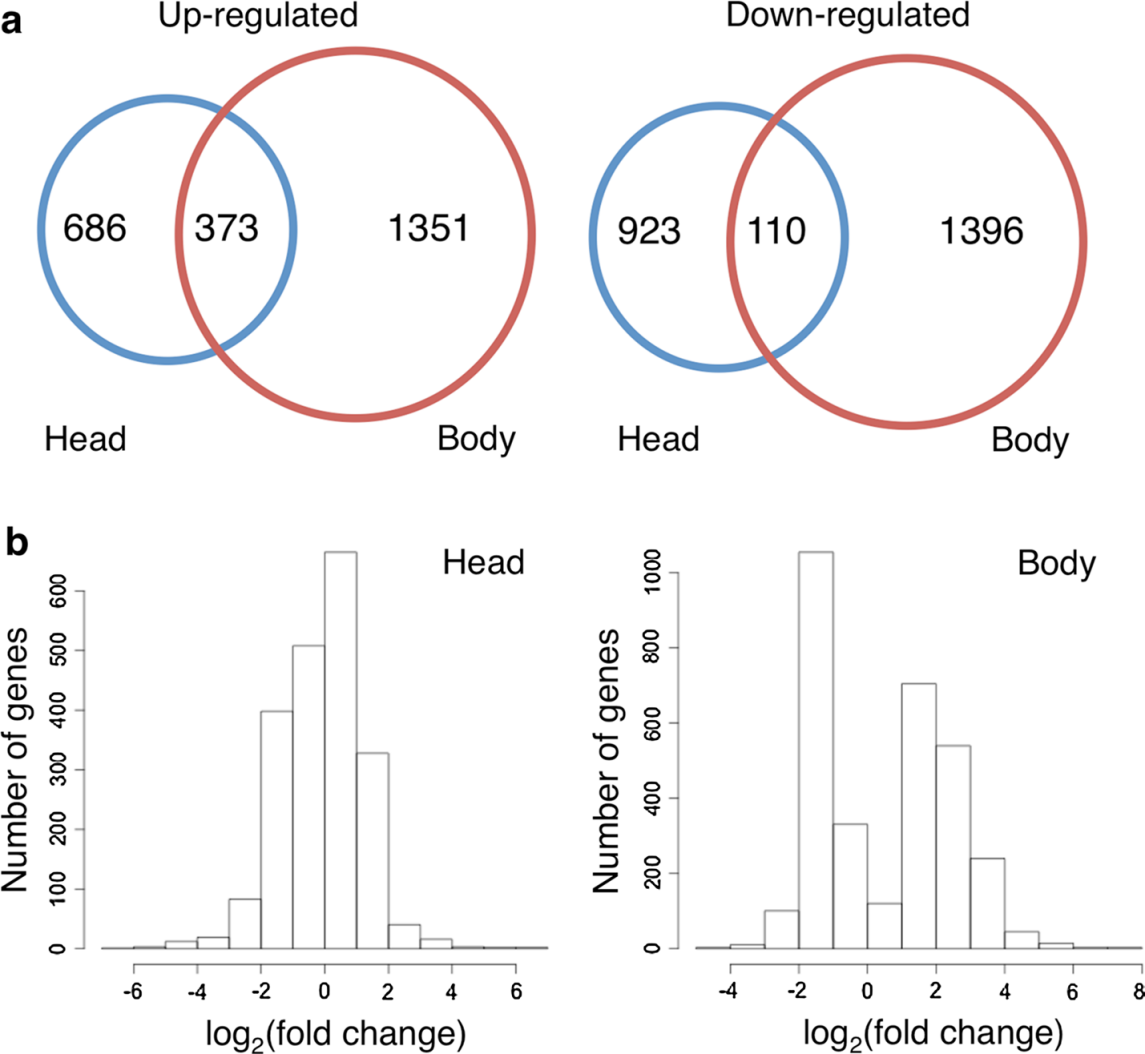
Summary of differentially expressed genes between summer and winter morphs of *D. suzukii*. **a** Venn diagram showing the number of up- and down-regulated genes in heads and bodies of winter morphs relative to summer morphs. **b** Histograms showing the distribution of fold changes of differentially expressed genes in summer and winter morphs. *Fold changes* represent the ratio of expression levels of winter to summer morphs. Genes are binned into groups based on log_2_ (fold change). Up- and down-regulated genes have positive and negative log_2_ (fold change) values respectively. *Left* and *right panels* show the histograms for head and body transcriptome data respectively. Histograms were generated using R. Cutoff q value (FDR-adjusted p value) < 0.05

To determine global gene expression differences that result in the morphological and physiological differences between summer and winter morphs of *D. suzukii*, we performed differential expression analysis using RNA sequencing between summer and winter morphs. Examination of gene expression in heads and bodies separately revealed a higher number of genes that are differentially expressed (up- or down-regulated) in bodies relative to heads [q value (FDR-adjusted p value) <0.05] (Figs. 5, 6a), even though the head and body transcriptomes contain similar number of genes that could be mapped to the reference genome (Additional file 2: Table S1). A scatter plot of FPKM values clearly illustrates that there are more differentially expressed genes (DEGs) between the two morphs in the body (Fig. 5b; Pearson’s correlation coefficient r = 0.6396) than in heads (Fig. 5a; Pearson’s correlation coefficient r = 0.9101).

**Fig. 7:**
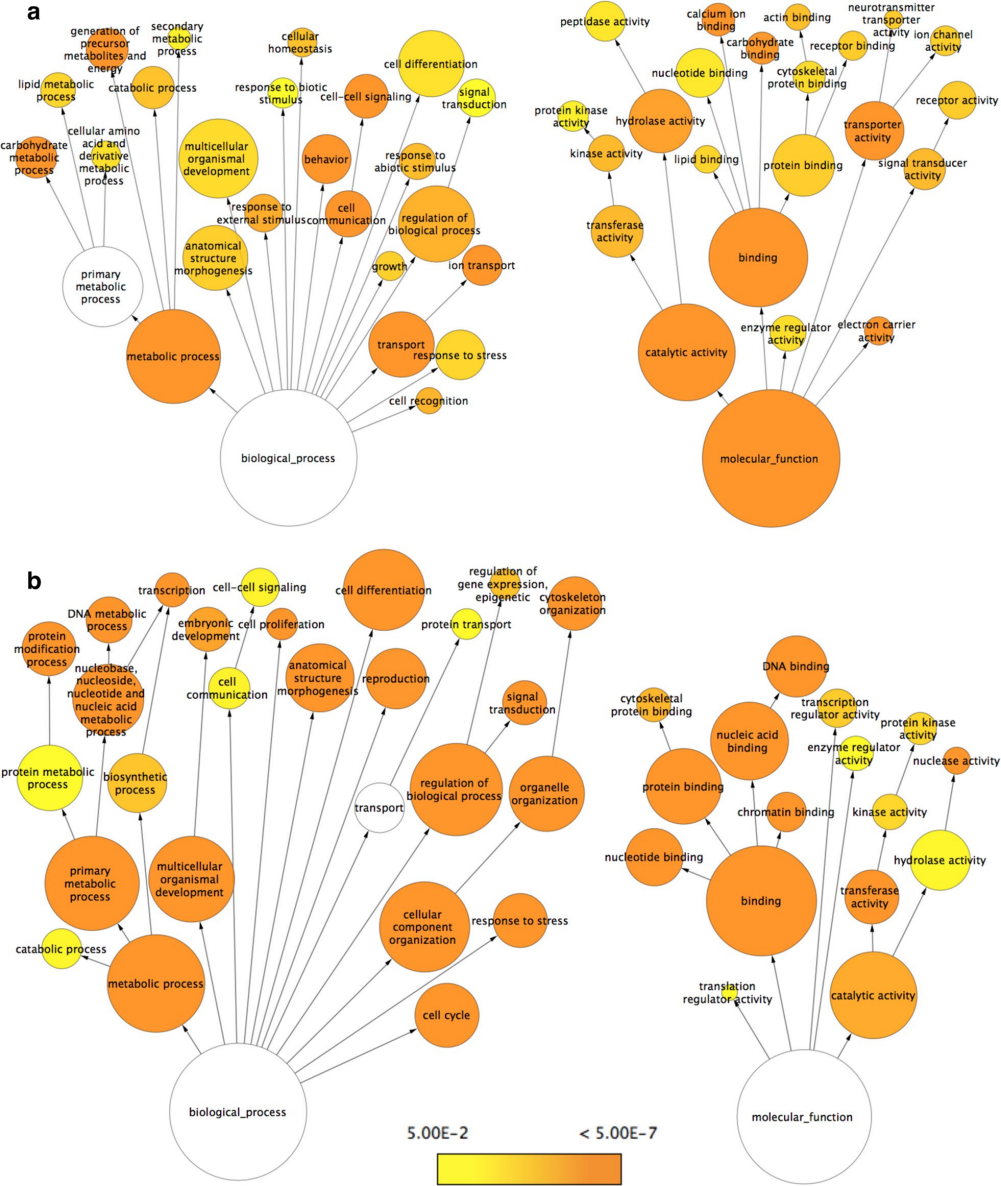
Cytoscape BiNGO visualization of overrepresented Gene Ontology (GO) categories in differentially expressed genes between summer and winter bodies of *D. suzukii* in the context of the GO hierarchy. Enriched GO terms that are (**a**) up-regulated and (**b**) down-regulated in winter bodies relative to summer bodies are classified by biological process (*left*) and molecular function (*right*). The size of each *circle* represents the number of genes that are included in each GO term and the *color of the circle* indicates the enrichment p value for the labeled GO term. As indicated in the enrichment scale, *orange* represents the highest enrichment and *yellow* represents the minimum enrichment above the cutoff (FDR corrected = 0.05). *White circles* represent nodes that are not enriched; they are shown in the figure to illustrate the GO term hierarchy and are only present if their “leaf nodes” are enriched. The hierarchical layout in Cytoscape was used to arrange the networks with manual adjustment of the nodes to allow for visualization of the text labels

**Fig. 8:**
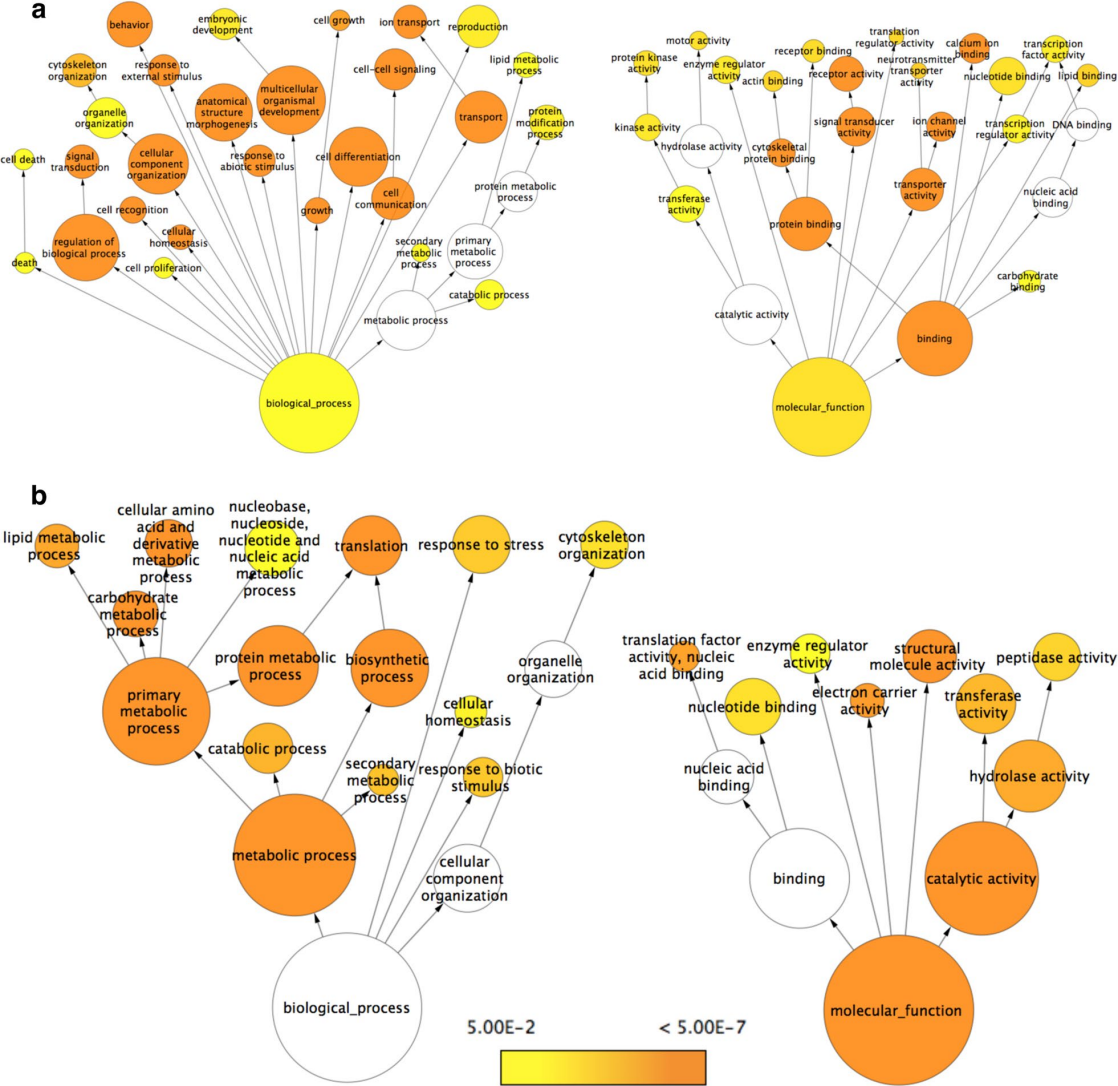
Cytoscape BiNGO visualization of overrepresented Gene Ontology (GO) categories in differentially expressed genes between summer and winter heads of *D. suzukii* in the context of the GO hierarchy. Enriched GO terms that are (**a**) up-regulated and (**b**) down-regulated in winter heads relative to summer heads are classified by biological process (*left*) and molecular function (*right*). The *size of each circle* represents the number of genes that are included in each GO term and the *color of the circle* indicates the enrichment p value for the labeled GO term. As indicated in the enrichment scale, *orange* represents the highest enrichment and *yellow* represents the minimum enrichment above the cutoff (FDR corrected = 0.05). *White circles* represent nodes that are not enriched; they are shown in the figure to illustrate the GO term hierarchy and are only present if their “leaf nodes” are enriched. The hierarchical layout in Cytoscape was used to arrange the networks with manual adjustment of the nodes to allow for visualization of the text labels

Moreover, the extent (fold change) of differential expression appeared much higher in bodies (Fig. 6b). It is possible that since the bodies contain some of the most metabolically active organs and tissues, e.g. fat body and muscles, many of the highly differentially expressed genes (DEGs) could be involved in the regulation of cellular metabolism, and possibly altered in winter morphs to enable winter survival. To systematically identify enriched categories of genes and molecular pathways that are differentially regulated between the summer and winter morphs, we performed Gene Ontology (GO) enrichment analysis using two independent methods, BiNGO 3.0.3 [54] and DAVID [55], which provided us with similar results. The output for BiNGO is presented in Figs. 7, 8, and the results from DAVID is presented in Additional files 3, 4, 5 and 6: Tables S2, S3, S4, and S5.

#### Up-regulated genes in bodies of winter morphs

The most significantly enriched terms in the winter bodies were those involved in glycolysis, the tricarboxylic acid (TCA) cycle, the electron transport chain, and ATP synthase (Fig. 7a and Additional file 3: Table S2). Based on the DAVID output, GO Terms involved with oxidative phosphorylation and the electron transport chain had the highest enrichment score, followed by glucose metabolism, TCA cycle, and terms involved with glycogen metabolism. Other notable enriched categories of genes that are up-regulated are involved in morphogenesis, development, and pigmentation, which is consistent with the enlarged, more melanized winter morph phenotype. Finally, genes involved in circadian rhythm and rhythmic behavior (e.g. *period*, *shaggy*, *timeless*) were also up-regulated in the bodies. There has been previous evidence suggesting that these genes are up-regulated in diapausing insects [25]. Although not listed as one of the enriched GO category, some of the most highly up-regulated genes in the winter bodies are genes involved in chitin biosynthesis and metabolism (Additional file 7: Table S6). The genes *CG14301*, *zye*, *kkv*, *Cpr76Bd*, *verm*, *Cpr47Ec*, *Cpr49Ae*, *obst*-*B*, and *Gasp* are all involved in chitin binding, structure, or metabolism and have a log_2_(fold change) greater than 5.0 in the winter bodies.

#### Down-regulated genes in bodies of winter morphs

The most significantly depleted terms in winter bodies were associated with the chromosome, chromatin organization, mitotic cell cycle, DNA replication, and DNA repair (Additional file 4: Table S3 and Fig. 7b). The enrichment score for most of these categories are very high, with many genes within these GO categories being down-regulated simultaneously. In addition, terms associated with the chorion, eggshell formation, oogenesis, and female meiosis were all enriched in down-regulated genes in the winter bodies. These results suggest a high likelihood that these female winter morphs are overwintering in reproductive diapause.

#### Up-regulated genes in heads of winter morphs

Based on DAVID output, the most significantly enriched GO terms were associated with immunoglobulin, plasma membrane, transmembrane, neuron development, ion transport, and muscle development (Additional file 5: Table S4). This is consistent with the BiNGO output, in which GO terms involved in ion channel activity, transporter activity, and multicellular organismal development are most enriched (Fig. 8a). In addition to being up-regulated in the body, genes involved in circadian rhythm were also up-regulated in the heads of winter morphs.

#### Down-regulated genes in heads of winter morphs

Some of the most significantly depleted terms in winter heads were those associated with the ribosome (high enrichment score), dehydrogenase, and organic acid biosynthesis (Additional file 6: Table S5). BiNGO also shows genes involved in carbohydrate metabolism, protein metabolic process, lipid catabolism, and translation being significantly down-regulated (Fig. 8b).

## Discussion

### *D. suzukii* can tolerate colder climates by transitioning into a winter morph

We first characterized seasonal morphs of *D. suzukii* in the field, and observed that wing length increased as winter approached and average temperature decreased. The proportion of flies with the winter morph phenotype also increased as the seasons progressed (Fig. 1). We then determined the abiotic factors needed to induce the winter morph phenotype that is observed in field-collected flies in the month leading up to winter. A laboratory-simulated winter-like photoperiod (12:12 L:D) and temperature (10 °C) was sufficient to induce higher levels of melanization and larger wing size. These changes in physiological characters are associated with an increase in cold hardiness as measured by longevity at 1 °C. While larger and darker forms of *D. suzukii* have been documented to occur in the fall [56], this is the first report characterizing intergeneration transition of *D. suzukii* seasonal morphs in field-collected populations resulting from environmental cues.

An increase in body size, using wing length as a proxy, in *D. suzukii* may be advantageous in colder environments, as it may aid in thermoregulation [44]. A larger body size may also allow for increased storage of sugars and fats, as overwintering insects often have an enlarged fat body [57]. However, a larger wing size may also be involved in increased dispersion capabilities. Interestingly, photoperiod and temperature appear to affect melanization in opposite ways. We found that shortening the photoperiod to a “winter” photoperiod, while keeping the temperature constant at 20 °C actually led to a decrease in melanization. This may be due to UV radiation protection; with a longer photoperiod, the flies are exposed to more light and therefore may need more melanin to protect from UV radiation [58]. Indeed, melanization of flies in sub-Saharan Africa most strongly correlate with UV radiation intensity when compared with other environmental factors, implicating a role for melanin in UV photoprotection [59]. Rearing the flies at a lower temperature of 10 °C significantly increases their melanization rating compared to flies reared at 20 °C. This suggests that melanization may play a role in cold tolerance in addition to its potential role in UV protection. An increase in melanization at low temperatures may increase UV absorption, increasing the ability to warm up. Further experiments are necessary to precisely identify the role of melanization in overwintering capability.

Past studies conducted on *D. suzukii* cold tolerance suggest that they have relatively low levels of cold tolerance [17, 18]. However, low levels of cold tolerance may represent a cost to improved plasticity [60]. In the experiments by Dalton et al. [17] and Jakobs et al. [18], flies were reared under summer conditions (25 °C) and then subjected to rapid or long-term cold-hardening. These conditions did not allow for developmental or intergeneration cold-hardening to occur, which is the focus of this current study. One recent study found that *D. suzukii* raised in simulated winter conditions has increased survival when briefly exposed to subzero temperatures, but this study did not investigate survival rates at prolonged periods of low temperature [53]. In our study, *D. suzukii* flies displaying the winter morph phenotype have low-temperature survival rates higher than those reported previously [17, 18]. In Dalton et al. [17], the LT_50_ period at 1 °C was 3 days within intra-generational acclimated adult flies, as opposed to the current study where the same level of mortality was reached at 115 days. These previous studies suggest that *D. suzukii* cannot survive winter conditions, but results presented in our study demonstrate that intergenerational and/or developmental plasticity in cold-tolerance may explain the presence of *D. suzukii* in cold northern locations. Our results support previous studies suggesting that *D. suzukii* overwinter in the adult stage [17, 29, 56, 61], and show that *D. suzukii* is more likely to successfully survive extended periods of cold in an adapted physiological state. Additionally, we show that male and female *D. suzukii* flies display differences in survival rates as measured by LT_50_. Stephens et al. [53] did not find any differences between sexes in supercooling points or lower lethal temperatures. Given our data, males may be more susceptible to cold, while females may preferentially survive the winter to reproduce in the spring. This is in agreement with the observation from Ometto et al. [16], suggesting a male bottleneck in *D. suzukii* population.

One aspect of current population modeling for *D. suzukii* that is significantly lacking is overwintering parameters. Our findings may be incorporated as seasonal parameters (e.g. [61]) in order to more accurately predict population levels and refine current management decisions. It is clear from phenological and physiological studies on *D. suzukii* [17, 61, 62] that winter is the bottleneck period for *D. suzukii* survival, as is the case for most insects. Knowledge of *D. suzukii* overwintering strategies and mechanisms is therefore of major importance when conducting risk assessment for the crop season immediately following the winter period.

It is known that *D. suzukii* is established in regions where temperatures frequently fall below freezing [13]. Although we did not test winter morph survival at subzero temperatures, Stephens et al. [53] predict that 50 % of *D. suzukii* adult summer and winter morphs die when exposed to approximately −10.01 and −15.3 °C, respectively. These basic physiological findings are not the only factors contributing to winter survival of *D. suzukii*. Behavior, suitable winter refuge sites, and suitable food sources will likely contribute to increased winter survival [63]. In addition, changes in humidity, which was not tested in our experiments but can be investigated in future experiments, may also be an environmental factor that trigger the transitions between summer to winter morphs and/or affect survival of seasonal morphs. [64]. Nevertheless, our findings greatly contribute to the understanding of *D. suzukii* overwintering mechanisms.

### Gene expression differences between summer and winter morphs of *D. suzukii* suggest altered metabolism and reproductive diapause in winter morphs

To begin to identify the molecular pathways that underlie the morphological and physiological differences observed in summer and winter morphs of *D. suzukii*, we performed global gene expression analysis using RNA sequencing. Among the many categories of DEGs, the biological processes that appear to be significantly altered in winter morphs of *D. suzukii* relative to the summer morphs include cellular metabolism, protein synthesis and translation, cell cycle and DNA replication, and chitin and cuticular protein synthesis.

#### Cellular respiration and metabolism

We found that genes involved in cellular respiration (i.e. glycolysis, TCA cycle, and electron transport chain) and glycogen metabolism were the most significantly enriched groups in genes that were up-regulated in winter morphs. This is somewhat surprising, as suppressed metabolism is a hallmark of diapausing insects [22]. However, up-regulation of glycolysis has previously been associated with an increase in polyol synthesis to yield increased level of cryoprotectants, and higher rates of anaerobic respiration in response to hypoxic conditions [37, 65]. These studies also found a decrease in TCA cycle enzymes, supporting the idea that anaerobic respiration dominates in some diapausing insects. However, many of these studies investigated insects that diapause in the pupal stage, which are more likely to overwinter in hypoxic conditions such as in soil. Since *D. suzukii* likely overwinter as adults, they may not be exposed to hypoxic conditions and therefore may be able to maintain a high rate of aerobic respiration as long as food resources are available. Indeed, in *D. melanogaster*, adult diapausing females had increased levels of glycolytic transcripts but also some increase in the TCA cycle transcripts [66].

Up-regulation of genes involved in cellular respiration may reflect the need for increased glycogen and fat stores. Genes involved with glycogen metabolism were highly enriched in bodies of winter morphs. Interestingly, both glycogen phosphorylase, which catalyzes glycogen breakdown, and glycogen synthase, which catalyzes glycogen synthesis, are both significantly up-regulated. The fact that both catabolic and anabolic enzymes are up-regulated may suggest a high rate of glycogen turnover. Futile cycling is a process by which two opposing pathways are active simultaneously where the only net effects are to hydrolyze ATP and to produce heat. Glycogen futile cycling has been observed in bacteria [67] and futile cycling has been found to produce significant amounts of heat in bumblebees [68]. High metabolic rates may be advantageous for ectotherms at low temperatures, as it may produce heat to raise body temperature. Alternatively, metabolic genes may also need to be up-regulated to compensate for lowered enzyme efficiency at lower temperatures. Since we are sequencing mRNA transcripts from entire bodies, the large transcript abundance of genes involved in cellular respiration may also arise from differences in tissue size. A highly enlarged fat body, as often seen in diapausing insects [57], may explain the enrichment of metabolic terms. For these reasons, physiological metabolic rate (as measured by heat rate, oxygen consumption, and carbon dioxide production) should be measured to further investigate overall metabolic differences between the summer and winter morphs.

Although our study focused on photoperiod and temperature as environmental cues to induce *D. suzukii* winter morph phenotype, other cues, such as food availability are likely important in regulating metabolism. Because our study did not address food scarcity issues that likely occur in the wild, further research is needed to determine metabolic gene expression differences in conditions that are more ecologically relevant.

#### Cell cycle, DNA replication, and protein synthesis

Genes involved in DNA replication, female meiosis, and egg production were highly down-regulated in the bodies of winter morphs, suggesting that they may be in reproductive diapause. Our gene expression data is consistent with studies in diapausing vs. nondiapausing *D. melanogaster* [66], showing down-regulation of similar gene classes in diapausing females. Suppression of DNA replication, growth, and decreased metabolic activity are hallmarks of diapause [21]. Increased metabolic rates, discussed earlier, may allow for accumulation of glycogen and/or lipid reserves that is associated with diapause.

Additionally, protein translation and ribosome biogenesis appeared to be substantially down-regulated in winter morphs of *D. suzukii*. This may be an adaptive mechanism that allows an insect to allocate energy and metabolites to more important processes such as increasing cold tolerance [69].

#### Chitin and cuticular protein synthesis

Among the over 1500 genes that were up-regulated in bodies of winter morphs, genes involved in chitin metabolism were the most highly up-regulated transcripts in terms of fold change. Increased chitin synthesis has been implicated in desiccation resistance [70]. These cuticular proteins may also be involved in repairing damage caused by desiccation [71]. An increase in cuticular lipids has been associated with decreased water loss and an increase in freeze-tolerance [72]. Finally, the increased expression of these genes may also be necessary for the large size of the winter morphs of *D. suzukii*. The fact that this class of genes are among the most up-regulated genes further warrant future investigation of humidity as a factor to trigger transition between summer to winter morphs and a variable that regulates survival of the seasonal morphs.

## Conclusions

In this study, we examined seasonal variations in morphology and physiology in *D. suzukii* and investigated the role of phenotypic plasticity in facilitating its rapidly expanding range. We investigated the role of temperature and photoperiod in the induction of seasonal phenotypic plasticity in this invasive species. Our study is a first step to better understand mechanisms employed by *D. suzukii* to survive harsh winter conditions and successfully expand its global range. Future work is necessary to determine the complex interaction of photoperiod, temperature, and other environmental cues, and the mechanisms by which they affect physiological responses and adaptation. In addition, experiments will be necessary to determine the developmental stage at which the cues need to be received and the mechanisms that enable transition between different phenotypic morphs.

Our gene expression analysis identified candidates that are important for the observed differences between the seasonal morphs and set the stage for functional characterization. In particular, many unannotated genes are highly expressed in the winter morph and have no known function. These genes may be involved in cold tolerance and/or diapause and could be of broad interest to the investigation of organismal physiology and adaptation. Moreover, transcript abundance is only one piece of the puzzle. Post-transcriptional and post-translational regulation of molecular pathways can certainly play additional roles in modulating the overall biochemical makeup of the organisms in response to environmental cues. Metabolomic profiling of *D. suzukii* summer and winter morphs can therefore provide further insight into biochemical mechanisms of increased cold tolerance and desiccation resistance.

*Drosophila suzukii* is emerging as a powerful model for ecological genetics due to its close phylogenetic relationship with the model organism *D. melanogaster*, its recently sequenced genome, and the expanding worldwide population monitoring and sampling network because of its economic importance as an agricultural pest. *Drosophila suzukii* is ideal for bridging the gap between laboratory model organisms for which molecular tools are readily available and ecological models used to study adaptations in natural environments.

## Methods

### Observation of phenology traits of *D. suzukii* in the field

Adult *D. suzukii* were captured in traps placed in the field (Hood River, OR, USA GPS coordinates 45°41′12.39′′ N 121°32′53.27′′ W) and were then measured for wing length and abdominal melanization (see below). Traps were constructed from clear 946 ml plastic food containers (Solo Cup Co., Lake Forest, IL). Each trap had 10–0.5 cm holes in the sides near the top. Traps were baited with 150 ml of clear apple cider vinegar and then capped. Approximately 1 ml of unscented dish soap (Ultra Pure Clear, Colgate-Palmolive Co., New York, NY) was added per liter of vinegar to break the surface tension. The vinegar attractant was replaced weekly. When present, adult *D. suzukii* were removed from the traps, sexed and placed in vials containing 70 % ethanol separated by collection date. Mean daily air temperatures were plotted from the Hood River, Oregon AgriMet Weather station (HOXO) [73]. Day-length values were plotted from the Astronomical Applications Department for the U.S. Naval Observatory for Hood River, Oregon [74].

### Measurement of abdominal melanization and wing length

Abdominal melanization was quantitatively rated using a visual scoring system under a stereomicroscope as described in [43, 72]. The thickness of each melanized band along the dorso-ventral line of abdominal segments 1–5 was estimated and scored on a scale from 0 (no melanization) to five (complete melanization) (Additional file 1: Figure S1). Melanization scores for the five melanized abdominal bands were compared between morphs for each sex.

The length of wings of field-collected *D. suzukii* as well as adults reared from day-length and temperature-controlled studies were measured as a proxy for body size as described in [75–77]. Wing size measurements were conducted on two segments along vein IV [75], [77, 78] of the left wing (Additional file 1: Figure S1). The first segment (L1) was measured from the base of the fourth longitudinal vein to the posterior cross vein. The second segment (L2) was measured from the posterior cross vein to the distal extreme of the fourth longitudinal vein. Wings were first dissected and then slide mounted in order to take digital photographs of the wings using a Leica camera (Leica DFC480, Buffalo Grove, Il) mounted on a binary microscope (Leica MZ12A, Buffalo Grove, Il). Images were then imported into imaging software (ImagePro Plus, MediaCybernetics, Rockville, MD) where length measurements were obtained.

### *Drosophila suzukii* strains and culture conditions

A *D. suzukii* stock colony was started from 200 individuals sourced from field collections during September 2012. All individuals were reared from wild blackberries, Rosaceae: *Rubus discolor*, collected in Hood River, OR USA (GPS coordinates 45°41′12.39′′ N 121°32′53.27′′ W). Permission for fly and blackberry collections as well as access to collection site was not required. Adult *D. suzukii* that emerged from the berries were placed in Bugdorm (299 × 299 × 299 cm, Model 1452, Bioquip, Rancho Dominguez, CA) rearing cages. These cages were modified by gluing clear plastic film (Flex-O-Glass, Warp Bros., Chicago, IL) over the screened walls to maintain humidity and prevent cross contamination. *D. suzukii* laboratory colonies were subsequently maintained at 23 ± 1 °C and a photoperiod of 16:8 Light:Dark (L:D) in hours.

Within the laboratory, *D. suzukii* was reared using a commercial *Drosophila* diet (Formula 4–24 Instant drosophila medium, Carolina Biological, Burlington, NC). Disposable polystyrene petri dishes (100 × 15 mm, VWR International, Radnor, PA) were filled with 100 ml of *Drosophila* diet and 100 ml of distilled water. A yeast paste (~1.5 ml) was made by mixing 15 g of yeast (Red Star, Lesaffre Yeast Corp., Milwaukee, WI) with 20 ml of water until a creamy consistency was achieved. The yeast paste was then applied as a thin strip to the top of the diet. Six petri dishes were added to each Bugdorm cage and left in the cage for 1 week to allow *D. suzukii* to oviposit. Each cage was also provided with three water containers with wicks and one 45 % sucrose solution (w/v) container with a wick. Petri dishes were replaced weekly and water and sugar water containers were refilled. Petri dishes removed from the cages were placed into clean Bugdorm cages to rear out future generations of *D. suzukii*. Once the next generation of *D. suzukii* adults began emerging, one petri dish with diet and yeast, water and sugar water were added to the cage. One week after the first *D. suzukii* adults emerged, petri dishes and additional pupae were removed from the cage in order to standardize the fly age to 1–7 day-old individuals. Six new petri dishes with diet and yeast were then added to the cage to begin rearing the next generation of *D. suzukii*. This process was repeated to maintain colonies for experiments.

*Drosophila suzukii* from the stock colony were reared in biological incubators (Model: l-36-LLVL, Percival, Perry, IA) under two regimes, 20 °C and 16:8 L:D photoperiod (“summer conditions”) and 10 °C and 12:12 L:D photoperiod (“winter conditions”) with relative humidity set to 70 %. The “summer” and “winter” photoperiod and temperature were chosen to reflect conditions in Hood River, OR in June and around October, when summer and winter morphs were observed respectively (Fig. 1). Temperature lower than 10 °C were not used due to the difficulty in rearing enough flies for experiments, and given the fact that winter morph phenotypes can be obtained with the simulated winter conditions we used. Adult *D. suzukii* that were maintained in simulated summer conditions (20 °C and 16:8 L:D) were transferred to specific test conditions upon emergence from pupae to allow for mating and seeding of the next generation of flies, and the morphology of the progenies were assessed. Rearing *D. suzukii* populations under the respective environmental conditions was conducted using clear polystyrene rearing vials (wide *Drosophila* vials, Genesee Scientific, San Diego, CA) capped with cellulose acetate plugs (Flugs, Genesee Scientific, San Diego, CA). Approximately 15 ml of water and 15 ml of *Drosophila* diet were added to each vial. After the water was completely absorbed by the diet, ~0.2 ml of yeast paste (see above) is added on top of the diet. Twenty-five adult male and female *D. suzukii* (F0 individuals) from the stock cultures each were added to vials and these adults were then left inside the vials under each of the four environmental conditions for 7 days in order to allow oviposition. The offspring from these individuals (F1 individuals) were transferred regularly to new vials containing a similar water/yeast mixture as described above. Various measurements of physiological traits were taken from parent flies (F0) and subsequent adult offspring.

### Examining the survival of summer and winter morphs at different temperatures

As *D. suzukii* adults emerged each week from rearing containers in the laboratory under either 20 °C and 16:8 L:D photoperiod or 10 °C and 12:12 L:D photoperiods, they were placed within vials containing optimized artificial rearing media based on their rearing environments. Vials contained five adult *D. suzukii* separated by sex and morph phenotype. These vials were then placed in one of five temperature and photoperiod regimes; 1 or 5 °C, both with 12:12 L:D photoperiod or 10, 20 and 28 °C with 16:8 L:D photoperiod. The survivorship of both male and female *D. suzukii* was determined within these vials by counting surviving individuals at 3–4 day intervals for a total observation period of 0–141 days. Eight replications, consisting of 1–3 vials of five 1–7 days-old flies of either sex for each of the five temperature regimes were used for this study. Data was averaged within a replicate if more than one vial of five flies was used. The length of time (d) to reach 50 % mortality (LT_50_) was estimated for each sex × morph × temperature replicate and then averaged to generate LT_50_ values for each combination of temperature, sex and morph phenotype.

### Statistical analyses

Differences in melanization ratings between seasonal morph phenotypes for various abdominal segments were analyzed with t tests for each sex. Wing length measurements for the two morphs were compared with two-way ANOVAs (GLM [79]). Paired t tests performed on estimated LT_50_ values (days) for each sex and morph at each temperature (ProcMeans [79]). A 1-tail paired t test was conducted to compare the LT_50_ of male and female winter morphs held at 1 °C. Wing length measurements were transformed using the square root before ANOVA to stabilize variances [80].

To identify the relationship between body size and two seasonally varying environmental factors, we performed linear regression analysis of wing length (i.e. a good proxy for body size) over temperature and day-length. Wing length data were generated by averaging individual wing length for each sex and collection date. We used the average daily temperature over the 12 days preceding the test date as a proxy for the temperature experienced by the developing fly. Temperature and day-length data were obtained from the Hood River, Oregon AgriMet Weather station (HOXO) [73] and the Astronomical Applications Department for the U.S. Naval Observatory [74].

### RNA extraction, transcriptome library preparation, and high-throughput sequencing

*Drosophila suzukii* used for transcriptome analysis were from the stock colony established from flies collected in Hood River, OR, U.S.A. Summer and winter morphs of *D. suzukii* were reared in simulated summer conditions: a photoperiod of 16:8 L:D and 20 °C and simulated winter condition: a photoperiod of 12:12 L:D and 10 °C respectively and were collected at age 4–5 days. Only females were included to control for sex-specific differences. Each biological replicate contained pools of 15 heads or bodies. Adult females were flash frozen on dry ice 4 h after lights on time and stored in 1.7 mL tubes (Denville, Holliston, MA) at −80 °C. Heads and bodies were separated on dry ice using metal sieves with 425 and 710 μm opening (Newark Wire Cloth Company, Clifton, NJ). To extract total RNA, tissues were first homogenized in 150 μL Tri Reagent (Sigma, St. Louis, MO) on ice using a motorized pestle (Kimble Chase, Vineland, NJ), and 350 μL Tri Reagent was subsequently added to bring the total volume to 500 μL. 100 μL chloroform was added, and tubes were inverted approximately 10 times. Samples were incubated at room temperature for 10 min and then centrifuged for 15 min at 13,000 RPM at 4 °C. Samples were placed on ice and 250 μL of the upper aqueous layer was transferred to a new 1.5 mL DNA LoBind tubes (Eppendorf, Hauppauge, NY). RNA was precipitated with 250 μL isopropanol (Sigma, St. Louis, MO) by incubating at −20 °C overnight. Samples were then centrifuged for 15 min at 13,000 RPM at 4 °C. The supernatant was removed and 800 μL 70 % ethanol was added to wash the RNA pellet. Samples were centrifuged for 5 min at 13,000 RPM at 4 °C and the ethanol was removed. The pellet was left to dry at room temperatures for 20 min. Head and body RNA samples were resuspended in 25 and 50 μL 1X TURBO DNase buffer, respectively. Each sample was treated with 1 μL TURBO DNase (Life Technologies, Carlsbad, CA). Samples were quantified using NanoDrop1000 (Thermo Scientific, Waltham, MA) and their quality assessed using the Experion Bioanalyzer (Bio-Rad, Hercules, CA). All RNA samples had an RNA Quality Indicator (RQI) >7.0. RNA sequencing libraries with insert size of approximately 150 bp were prepared using 1 μg total RNA with the Illumina TruSeq RNA Sample Preparation kit according to manufacturer instructions (Illumina, San Diego, CA). Libraries were submitted to BGI Americas (Sacramento, CA, USA) where library size and quality was assessed using an Agilent 2100 Bioanalyzer (Agilent Technologies, Santa Clara, CA). Samples were quantified using quantitative PCR, pooled, and sequenced on the Illumina HiSeq 2000 using paired-end 100 bp sequencing. Eleven RNA sequencing libraries were prepared in total: three biological replicates each for summer heads, summer bodies, and winter heads, and two biological replicates for winter bodies.

### Transcriptome assembly, identification of differentially expressed genes (DEGs), and Gene Ontology (GO) enrichment analysis

We generated a total of 309 million paired-end reads from 11 libraries. We performed biological triplicates for all treatments, except for bodies of winter morphs, for which biological duplicates were used. Raw reads were mapped to the *D. suzukii* reference genome [81] using Bowtie-based Tophat v2.0.12 [82], resulting in an average of 20.5 million mapped reads per replicate (Additional file 2: Table S1). Pearson correlations of expression levels in FPKM between biological replicates were computed in R (Additional file 8: Table S7). Cufflinks v2.2.1 was used to identify differential expressed (DE) genes (q value (FDR-adjusted p value) < 0.05) [82], and CummeRbund [83] was used to visualize the results (Additional File 7: Table S6; and Additional file 9: Table S8). Up- and down-regulated genes were extracted from the list of DEGs, and analyzed for Gene Ontology (GO) enrichment using the BiNGO 3.0.3 [54] plug-in in Cytoscape. Hypergeometric test with Benjamini and Hochberg False Discovery Rate correction for multiple testing was used to access overrepresentation of generic GO slim terms for each condition. GO annotation of *D. melanogaster* orthologs was obtained from FlyBase FB2015_02 release. BiNGO visualization of overrepresented biological process and molecular function GO categories for body and head are shown in Figs. 7, 8 respectively. Independently, we also used the Functional Annotation Clustering tool in DAVID [55] to perform enrichment analysis and clustering, results of which are presented in Additional files 3, 4, 5, 6: Tables S2, S3, S4, S5.

### Availability of supporting data

The RNA sequencing data sets supporting the results of this article are available in the National Center for Biotechnology Information (NCBI) repository. [BioProject PRJNA294845 http://www.ncbi.nlm.nih.gov/bioproject/ PRJNA294845/, and Sequence Read Archives SRS1057327 (summer bodies) http://www.ncbi.nlm.nih.gov/ sra/?term = SRS1057327/; SRS1057275 (summer heads) http://www.ncbi.nlm.nih.gov/ sra/?term = SRS1057275/; SRS1057328 (winter bodies) http://www.ncbi.nlm.nih.gov/ sra/?term = SRS1057328/; SRS1057296 (winter heads) http://www.ncbi.nlm.nih.gov/ sra/?term = SRS1057296/]. NCBI accession for individual replicates are also provided in Additional file 2: Table S1.

## Additional files

10.1186/s12898-016-0070-3 Measurements of abdominal melanization and wing length in adult *D. suzukii*. (a) The thickness of the dark abdominal bands was used to differentiate the lighter colored summer morph (left) from darker winter morph (right) of *D. suzukii.* (b) Locations (L1 and L2) where wing measurements were taken from the excised left wings of *D. suzukii.*

10.1186/s12898-016-0070-3 Number of mapped reads and NCBI Genbank Accession number for each biological replicate of all four treatments reported by Tophat. S = Summer; W = Winter; H = Head; B = Body. Three biological replicates were performed for each treatment, except for fly bodies of winter *D. suzukii* morphs (WB), which has two replicates.
10.1186/s12898-016-0070-3 Functional Annotation Clustering of genes that are significantly up-regulated in the winter fly bodies relative to summer bodies as determined by DAVID.
10.1186/s12898-016-0070-3 Functional Annotation Clustering of genes that are significantly down-regulated in the winter fly bodies relative to summer bodies as determined by DAVID.
10.1186/s12898-016-0070-3 Functional Annotation Clustering of genes that are significantly up-regulated in the winter fly heads relative to summer heads as determined by DAVID.
10.1186/s12898-016-0070-3 Functional Annotation Clustering of genes that are significantly down-regulated in the winter fly heads relative to summer heads as determined by DAVID.
10.1186/s12898-016-0070-3 Table of differentially expressed genes in bodies of winter morphs of *D. suzukii* relative to those of summer morphs. Fold change represents the ratio of expression levels of winter to summer morphs.
10.1186/s12898-016-0070-3 FPKM correlation matrix for (A) head and (B) body RNA-seq replicates. Three replicates were performed for each condition, except for winter-body (WB), which has two replicates. (S = Summer; W = Winter; H = Head; B = Body).
10.1186/s12898-016-0070-3 Table of differentially expressed genes in heads of winter morph of *D. suzukii* relative to those of summer morphs. Fold change represents the ratio of expression levels of winter to summer morphs.

## Abbreviations

DEG: differentially expressed genes
FPKM: fragments per kilobase of transcript per million mapped reads
FDR: false discovery rate
GLM: general linear model
GO: gene ontology
L:D: light:dark
LT_50_: lethal temperature at which 50 % of the individuals perish
