# Supplementary figures and images for: Seasonal cues induce phenotypic plasticity of Drosophila suzukii to enhance winter survival
Source: BMC Ecol. 2016 Mar 22;16:11. doi: 10.1186/s12898-016-0070-3 (PMC4802914; doi:10.1186/s12898-016-0070-3)

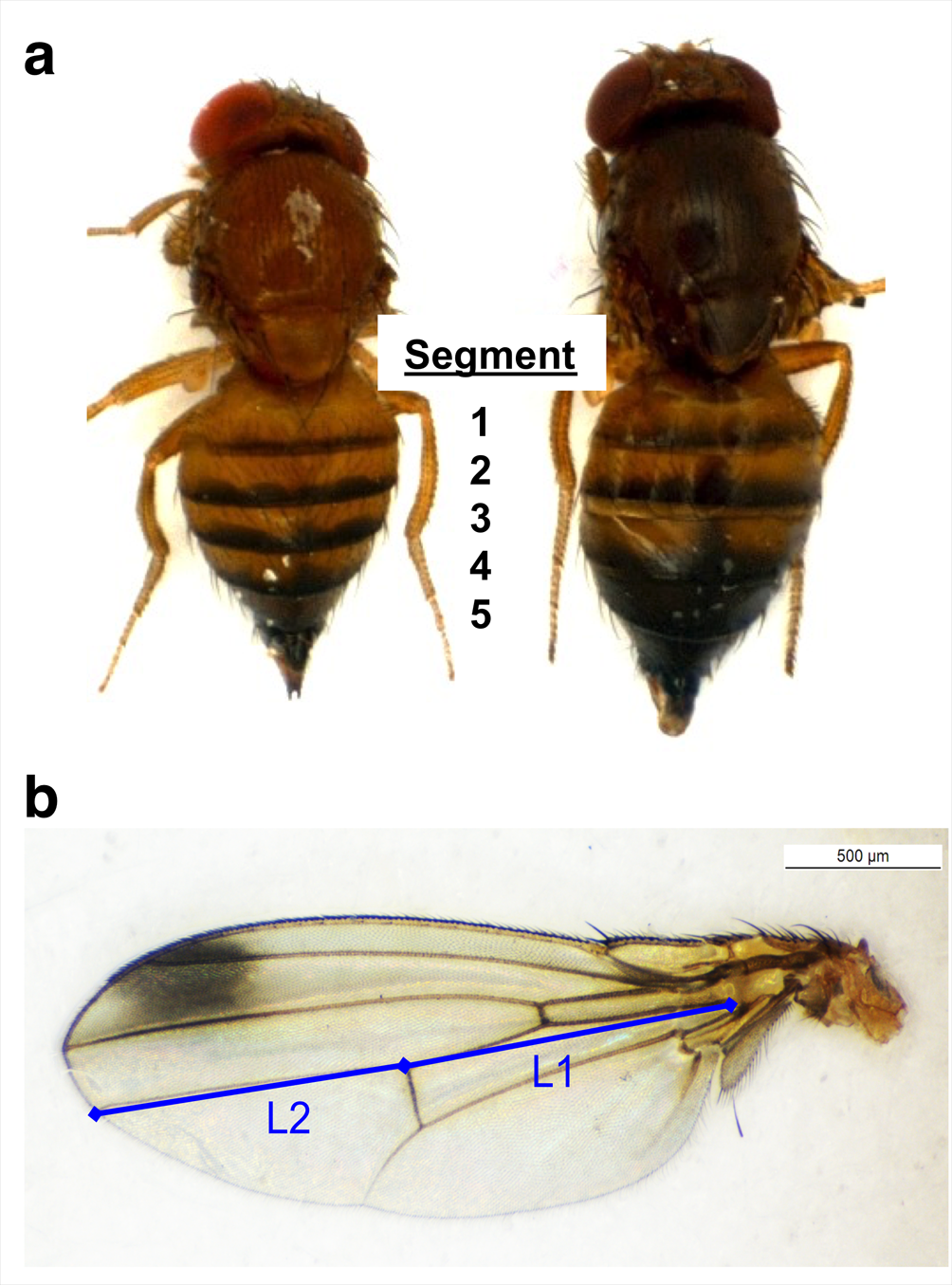

Supplement: Supplementary file 1 — 10.1186/s12898-016-0070-3 Measurements of abdominal melanization and wing length in adult D. suzukii. (a) The thickness of the dark abdominal bands was used to differentiate the lighter colored summer morph (left) from darker winter morph (right) of D. suzukii. (b) Locations (L1 and L2) where wing measurements were taken from the excised left wings of D. suzukii. [file 12898_2016_70_MOESM1_ESM.tiff]
