# Supplementary material for: Seasonal cues induce phenotypic plasticity of Drosophila suzukii to enhance winter survival
Source: BMC Ecol. 2016 Mar 22;16:11. doi: 10.1186/s12898-016-0070-3 (PMC4802914; doi:10.1186/s12898-016-0070-3)
Supplement: Supplementary file 2 — 10.1186/s12898-016-0070-3 Number of mapped reads and NCBI Genbank Accession number for each biological replicate of all four treatments reported by Tophat. S = Summer; W = Winter; H = Head; B = Body. Three biological replicates were performed for each treatment, except for fly bodies of winter D. suzukii morphs (WB), which has two replicates. [file 12898_2016_70_MOESM2_ESM.docx]

**Table S1. Number of mapped reads and NCBI SRA accession number for each biological replicate of all four treatments reported by Tophat.** S=Summer; W=Winter; H=Head; B=Body. Three biological replicates were performed for each treatment, except for fly bodies of winter *D. suzukii* forms (WB), which has two replicates.

| **Replicates** | **SRA Accession** | **No. of Mapped Reads** |
| --- | --- | --- |
| SB0 | SRX1193120 | 17,226,968 |
| SB1 | SRX1193121 | 19,382,542 |
| SB2 | SRX1193122 | 17,845,249 |
| SH0 | SRX1192626 | 27,104,580 |
| SH1 | SRX1192815 | 14,719,252 |
| SH2 | SRX1192816 | 23,070,263 |
| WB0 | SRX1193123 | 24,036,225 |
| WB1 | SRX1193124 | 16,918,209 |
| WH0 | SRX1192817 | 24,147,286 |
| WH1 | SRX1192882 | 20,213,964 |
| WH2 | SRX1192883 | 20,845,574 |
