# Supplementary material for: Seasonal cues induce phenotypic plasticity of Drosophila suzukii to enhance winter survival
Source: BMC Ecol. 2016 Mar 22;16:11. doi: 10.1186/s12898-016-0070-3 (PMC4802914; doi:10.1186/s12898-016-0070-3)
Supplement: Supplementary file 8 — 10.1186/s12898-016-0070-3 FPKM correlation matrix for (A) head and (B) body RNA-seq replicates. Three replicates were performed for each condition, except for winter-body (WB), which has two replicates. (S = Summer; W = Winter; H = Head; B = Body). [file 12898_2016_70_MOESM8_ESM.docx]

**Table S7**. **FPKM correlation matrix for (A) head and (B) body RNA-seq replicates.** There are three replicates for each condition, except for winter-body (WB), which has 2 replicates. (S=Summer; W=Winter; H=Head; B=Body)

(A)

|  | SH0 | SH1 | SH2 | WH0 | WH1 | WH2 |
| --- | --- | --- | --- | --- | --- | --- |
| SH0 | 1.0000000 |  |  |  |  |  |
| SH1 | 0.9728814 | 1.0000000 |  |  |  |  |
| SH2 | 0.8881385 | 0.8566596 | 1.0000000 |  |  |  |
| WH0 | 0.8632906 | 0.8702675 | 0.8828334 | 1.0000000 |  |  |
| WH1 | 0.7980491 | 0.7629274 | 0.7902504 | 0.9334621 | 1.0000000 |  |
| WH2 | 0.8787966 | 0.9153980 | 0.8517279 | 0.9350631 | 0.8315478 | 1.0000000 |

(B)

|  | SB0 | SB1 | SB2 | WB0 | WB1 |
| --- | --- | --- | --- | --- | --- |
| SB0 | 1.0000000 |  |  |  |  |
| SB1 | 0.8554288 | 1.0000000 |  |  |  |
| SB2 | 0.9470172 | 0.9041447 | 1.0000000 |  |  |
| WB0 | 0.5642950 | 0.5418976 | 0.5256237 | 1.0000000 |  |
| WB1 | 0.6158217 | 0.6826467 | 0.6304389 | 0.8910718 | 1.0000000 |
